# Evaluating machine learning algorithms at predicting developmental trajectories using sequential dataset truncation of voluntary alcohol consumption in adolescent mice

**DOI:** 10.1371/journal.pone.0352197

**Published:** 2026-06-22

**Authors:** Nathan Yu, Steven Buyske, Uthman Qureshi, Lei Yu

**Affiliations:** 1 Department of Genetics, Center of Alcohol & Substance Use Studies, Rutgers University, Piscataway, New Jersey, United States of America; 2 Department of Statistics, Center of Alcohol & Substance Use Studies, Rutgers University, Piscataway, New Jersey, United States of America; 3 Center of Alcohol & Substance Use Studies, Rutgers University, Piscataway, New Jersey, United States of America; The University of Sheffield, UNITED KINGDOM OF GREAT BRITAIN AND NORTHERN IRELAND

## Abstract

**Background:**

Adolescent alcohol consumption is a known risk factor for developing alcohol use disorder (AUD) in adulthood, but individual susceptibility varies widely, contributed to by differences in factors that are not well-understood. Identifying patterns of developmental trajectories in voluntary alcohol consumption behavior during adolescence could provide insight into biological underpinnings of AUD risk. Machine learning (ML) offers powerful pattern recognition capabilities that may help forecast future behavioral trajectories based on early-stage data.

**Objective:**

This study aimed to evaluate the performance of twelve supervised ML algorithms in predicting developmental trajectories of voluntary alcohol consumption behavior in adolescent mice using sequentially truncated datasets.

**Methods:**

Simulated balanced datasets of alcohol consumption in adolescent mice were generated based on previously published biological data. We applied a sequential dataset truncation strategy to train and evaluate ML models on progressively longer spans of behavioral data. Prediction accuracy for trajectory pattern classification was assessed for each truncation point, and goodness-of-fit was modeled using four curve-fitting equations, including locally estimated scatterplot smoothing (LOESS), which provided best fit and was selected for downstream comparative analysis.

**Results:**

LOESS-fitted accuracy progression curves enabled quantitative comparison across models. Six ML algorithms—Random Forest, Logistic Regression, Multilayer Perceptron, Linear Discriminant Analysis, K-Nearest Neighbors, and Support Vector Machine—achieved outstanding results, with 98% or better prediction accuracy by experiment end and 90% or better accuracy at midpoint. Four additional algorithms—Stochastic Gradient Descent, Decision Tree, Gradient Boosting Classifier, and Multinomial Naive Bayes—achieved acceptable accuracy values (77–95% at midpoint, and 91–96% at experiment end). In contrast, two models (Quadratic Discriminant Analysis and Gaussian Process Classifier) performed poorly and displayed declining accuracy trends with more data.

**Conclusions:**

This study demonstrates that certain supervised ML algorithms can accurately predict behavioral outcomes from early-stage data. This approach holds promise for guiding molecular and cellular analyses at time points prior to behavioral phenotype’s fully manifesting, making it possible to identify potential biological drivers that initiate the onset of harmful behavior of alcohol consumption during adolescence development.

## 1. Introduction

Adolescence is a developmental period marked by profound and stressful physical, behavioral, and cognitive changes [1–7]. Alcohol consumption during adolescence can lead to AUD later in life [8–18], contributing to the substantial societal and personal costs of AUD [19–21]. However, not everyone with alcohol exposure during adolescence is at equal risk. In humans, prevalence of exposure to alcohol during adolescence — often in social or celebratory contexts — is estimated at around 33% in youth aged 12–20 in the United States [22,23], and at approximately 29% to 91% for 15–16-year-olds across European countries [24,25]. Yet, AUD prevalence is estimated at approximately 11–14% for lifetime diagnosis in U.S. adults [16,26], indicating that the majority of the population, despite access and prior exposure to alcohol, does not develop AUD. Studies strongly support the combined contributions of genetic, epigenetic, and environmental factors to addiction vulnerability [26–30]. To study the factors and biological mechanisms that lead to high alcohol consumption behavior, it will be helpful to model various developmental trajectories from initial alcohol exposure during adolescent to adulthood status regarding alcohol consumption.

Rodent models make it possible to set up experimental studies of alcohol consumption behavior, including allowing initial alcohol exposure in adolescent animals, and analyzing their developmental trajectories in voluntary alcohol consumption behavior. In the spirit of humane experimental use of lab animals (replacement, reduction, refinement) [31,32], with an effort to more fully utilize animal research data, we applied finite mixture modeling, and showed that developmental trajectories are highly stylized for voluntary alcohol consumption in mice [33], with stable outcome status for adult mice regarding their alcohol consumption behavior, similar to the persistent nature of AUD patterns in humans. This finding prompts a tantalizing question: would it be possible to predict alcohol consumption behavior early on in life, before AUD behavioral patterns set in? A confirmative answer would open the door for testing early interventional measures that may influence the future outcome before a harmful AUD pattern is established.

Our previous findings [33] highlight a phenomenon: exploratory studies in biomedical research, particularly those involving longitudinal behavioral measurements, are often conducted with relatively modest sample sizes, yet may lead to meaningful observations of novel patterns in subgroups. Indeed, small-sample observations have historically played an important role in identifying novel phenotypes and disease entities, particularly in early-stage or hypothesis-generating research, such as Alzheimer’s description of a disease in a single patient [34,35], and Parkinson’s clinical description of a disease based on six patients [36,37]. Thus, even though distinct behavioral subgroups may comprise only a small fraction of the overall sample, their identification and characterization can still provide significant value, as it enables the formulation of new hypotheses regarding disease progression or behavioral regulation. Therefore, exploratory findings can, in turn, guide the design of future studies with larger and more targeted cohorts, facilitating rigorous validation and extension of initially observed phenomena.

To be able to identify and interrogate behavioral patterns in longitudinal studies, analytical approaches must accommodate heterogeneity across individuals while remaining effective in modeling various patterns in datasets. ML offers a robust approach for pattern recognition [38–40], and therefore an opportunity to predict the eventual outcome of certain patterned behaviors. In the present study, we evaluated supervised ML algorithms for their ability to predict developmental trajectories of voluntary alcohol consumption behavior in adolescent mice, using sequentially truncated data to measure ML prediction accuracy. We report the findings here.

## 2. Materials and methods

### 2.1. Animals and ethics statement

Data used in this study were from previously described work in mice [33,41–43]. Briefly, male outbred mice (3 weeks old) were group-housed in temperature controlled animal facilities on a 12 hr:12 hr light-dark cycle with food and water available ad libitum. When the mice were used in the chronic alcohol drinking experiment, they were singly housed and had 2 days of acclimation before starting the experiment. Principles of laboratory animal care were followed, and all procedures were approved by the Institutional Animal Care and Use Committee (IACUC) at the Chinese Academy of Sciences, Shanghai, China, and were performed in accordance with the National Institutes of Health (NIH) Guide for the Care and Use of Laboratory Animals and the Animal Welfare Act.

### 2.2. Voluntary alcohol consumption dataset from adolescent mice, and data simulation

As we reported previously [41], for the voluntary alcohol drinking experiment, the paradigm of two-bottle free-choice was used [44–49], with each cage equipped with two drinking tubes. Standard rodent chow was available ad libitum, and was spread around both drinking tubes to avoid food-associated tube preference. Animals’ body weight and food consumption were measured daily on weekdays. Voluntary alcohol consumption from adolescent mice was analyzed and reported previously as weekly aggregated data [33]. For the current study, however, daily alcohol intake data were used to assess sequential prediction accuracy values of various ML algorithms.

The original biological cohort consisted of 35 adolescent mice. Mice at 3 weeks old were acclimated to the housing for a week. At the beginning of the voluntary alcohol consumption study, mice were 4-weeks old, somewhat comparable to pre-adolescence in human development [5,6,50–53]. Except for daily weighing, no other handling or behavioral tests were performed during the voluntary alcohol consumption period until mice reached 11 weeks of age, comparable to young adulthood in human development [5,6,50–53]. Alcohol consumption behavior in these mice falls into one of three distinct drinking trajectory patterns: non-drinkers, late drinkers, and early drinkers, reported previously [33]. Daily alcohol consumption values of the mice were used in simulation: for each drinking subgroup’s daily alcohol consumption values listed in Excel, 50 simulated drinking values were generated in a normal distribution with 10% variability, for that behavior subgroup for that experimental day. Thus, a simulated dataset of 150 mice was generated, 50 for each of the three drinking trajectory subgroups.

### 2.3. ML models

The original experiment with adolescent mice spanned 52 time points (experimental days) of alcohol consumption measurements, covering the developmental period from adolescence-to-adulthood [5,6,50–53]. Using simulated data, each trajectory was truncated to include only the initial time point (Day 1 only) to construct the first dataset. Subsequently, one additional day of data was added to construct the next dataset, and the process was repeated until the full duration of the longitudinal dataset (Day 1–52) was constructed.

ML models for classification were selected according to a literature review of reputable and effective algorithms [54–56], and all models were implemented using the library Scikit-learn. To facilitate ready adoption by other users, whenever possible, default parameters and implementation were used as provided by Scikit-learn. All results are normalized on a scale of 0–1. Details about parameters and tuning are listed in the supporting materials file (S3 Data).

Each dataset of 150 instances (three alcohol consumption subgroups, 50 per subgroup) was split into 70% for training and 30% for evaluation. For each of the 52 truncated datasets (52 experimental days), a new split was independently generated with the 70/30 training/testing ratio. All ML algorithms were then performed on the same split, and their accuracy values for trajectory subgroup classification recorded for that time point. Twelve algorithms were tested. These include: Random Forest, Logistic Regression, Multilayer Perceptron, Linear Discriminant Analysis, K-Nearest Neighbors, Support Vector Machine, Stochastic Gradient Descent, Decision Tree, Gradient Boosting Classifier, Multinomial Naive Bayes, Quadratic Discriminant Analysis, and Gaussian Process Classifier.

### 2.4. Statistics

ML algorithm accuracy values were smoothed by LOESS [57], and values at the midpoints and endpoints were extracted. LOESS smoothing was performed using the LOESS 2.1.2 package for Python, parameters and variables are included in supporting materials (S2 Data). Mean absolute error (MAE) from the LOESS fit at the midpoint and endpoints of the experiment were used as summary statistics for the various ML algorithms. GraphPad Prism software was used (version 9.5, GraphPad Software, San Diego, CA, USA), with significance set at an alpha level of 0.05.

## 3. Results

### 3.1. Voluntary alcohol consumption in adolescent mice: Balanced datasets of mouse behavior for use in training and evaluating ML algorithms

We previously reported that, for voluntary alcohol consumption behavior in adolescent mice, there are three distinct developmental trajectories [33]. These trajectories—early drinkers, late drinkers, and non-drinkers—highlighted individual differences in alcohol intake throughout the adolescent-to-adulthood development. The clear separation of these behavioral patterns suggests that each behavior trajectory may reflect distinct underlying biological mechanisms, making them an attractive use case for utilizing ML-based prediction models.

**Fig 1:**
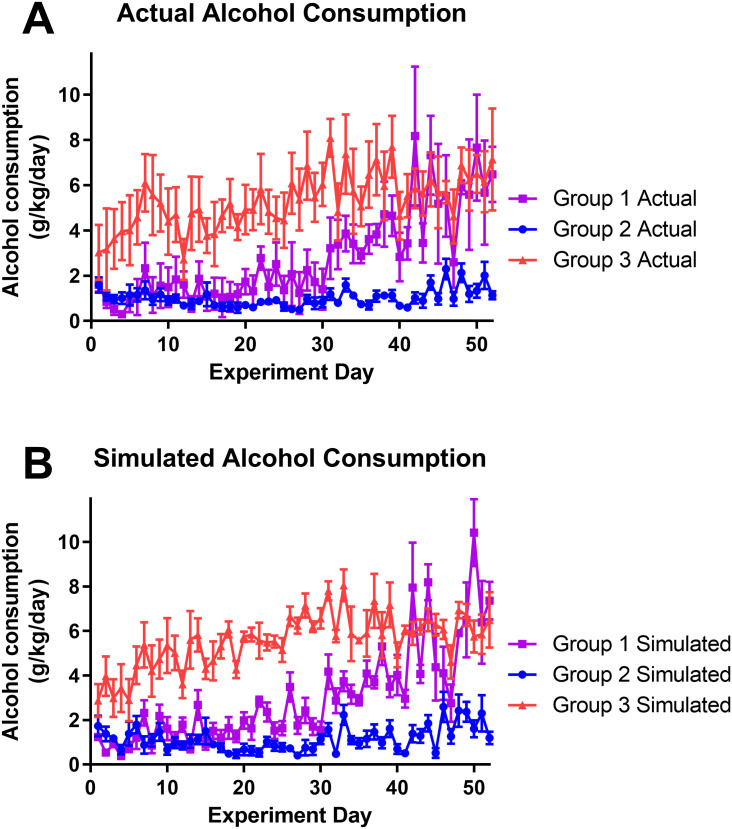
Developmental trajectories of voluntary alcohol consumption in adolescent mice, showing similar patterns of actual and simulated data. (A) Actual alcohol consumption data: the three distinct behavioral trajectory patterns of voluntary alcohol consumption in adolescent mice (mean ± SEM; n = 4 for Group 1 “late drinkers,” 26 for Group 2 “non-drinkers,” and 5 for Group 3 “early drinkers”). (B) Simulated alcohol consumption data: three subgroups of simulated alcohol consumption (mean ± SEM; n = 10 for each subgroup).

This approach, while seemingly desirable, presents a challenge: ML algorithms usually train on balanced datasets, i.e., the numbers for various target classes tend to have similar ratios. A major limitation of the original dataset is its class imbalance. As shown in Fig 1A, while all three behavioral trajectories are distinct, the majority of mice fall into the non-drinker subgroup (“Group 2,” n = 26), with far fewer mice being in the late drinkers (“Group 1,” n = 4) or the early drinkers (“Group 3,” n = 5). While the lopsided number distribution among the three classes may well reflect the biological reality (in humans, individuals with AUD represent a small percentage of the general population, with the majority being “non-drinkers” [16,26]), typically supervised ML algorithms are trained on relatively balanced data classes, and highly skewed class distributions of imbalanced datasets require specialized methodological handling [58–62].

To address this issue, we generated a simulated dataset of alcohol consumption patterns. As an exploratory study to evaluate the utility of using ML algorithms for longitudinal trajectory prediction, our intent in data simulation is to provide controlled, reproducible input datasets for evaluating ML algorithm performance, and to systematically assess robustness under conditions of simulated data based on the original limited and imbalanced biological data. Thus, the purpose of simulation is not focused on biologically valid extrapolation, but rather using simulated data for stress-testing ML model capabilities. Under such considerations, the intent is more concerned with simulated data approximating the overall structure of the limited biological datasets, rather than about whether the simulated data fully represents biological variability. In this context, potential overfitting or ML algorithm sensitivity to small samples becomes a factor under active evaluation. For assumption to be used in simulation, we considered the fact that many biological measurements are often approximately normally distributed, as they arise from the additive effects of multiple independent factors, consistent with the Central Limit Theorem [63–65]. However, we do recognize that deviations from normality are common, as biological data may exhibit skewness, multimodality, or other non-Gaussian features [65]. Since the focus of this study is to test ML algorithm capabilities, not on data distribution per se, we used Excel to generate simulated data in a normal distribution with 10% variability, with 50 simulated values of mouse alcohol consumption for each of the experimental days, and for each of the three subgroups, with a total of 150 simulated longitudinal datasets. Therefore, the simulated datasets are modeled after the original biological data, but structured to be numerically balanced across the three behavioral subgroups (data provided in S1 Data).

As previously reported [33], three distinct developmental trajectory patterns (subgroups) were identified based on their voluntary alcohol consumption behavior: “non-drinkers” (“Group 2”), “late drinkers” (“Group 1”), and “early drinkers” (“Group 3”). Non-drinkers exhibited consistently low alcohol consumption throughout the study, late drinkers did not consume alcohol in early adolescence but showed a significant increase in alcohol intake during adolescence-to-adulthood transition, and early drinkers maintained high levels of consumption from the start. These behavioral patterns are clearly displayed in the original 35 mouse dataset (Fig 1A). For simulated data, as an illustrative example, Fig 1B shows a randomly sampled subset of 10 simulated mice per subgroup, showing that the developmental patterns of alcohol consumption closely mirror those of the original biological dataset (Fig 1A).

This balanced and scalable synthetic dataset now appear to preserve the developmental trajectory features identified in the original experimental subgroups, therefore providing an expanded and uniformly sized data sample suitable for training and evaluating ML algorithms. By mitigating the effects of class imbalance, the simulated dataset enables a more reliable ML model evaluation.

### 3.2. Using sequential dataset truncation to train and evaluate ML algorithms

**Fig 2:**
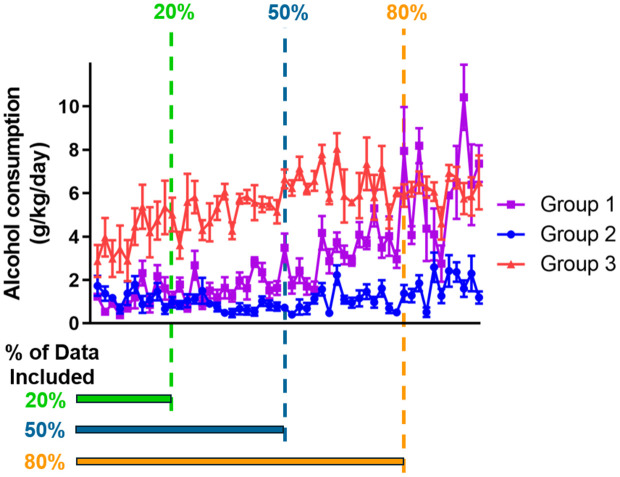
Schematic diagram showing the principle of sequential dataset truncation. Alcohol consumption trajectories are shown for three subgroups (Group 1: late drinkers; Group 2: non-drinkers; Group 3: early drinkers). Vertical dashed lines represent example truncation points at 20%, 50%, and 80% of the data, respectively. For each truncation level, the truncated dataset is used to train and evaluate ML models with a 70/30 split. This approach allows evaluation of model prediction accuracy for trajectory subgroup classification over different data spans of the adolescent developmental period.

To assess the ability of ML algorithms to predict the future behavioral trajectory of voluntary alcohol consumption in adolescent mice, we employed a sequential dataset truncation strategy. This method is conceptually illustrated in Fig 2. The idea is to iteratively train and evaluate ML models using progressively longer spans of time-series behavioral data, thereby simulating the real-world need of predicting future outcomes from early behavioral indicators.

In practice, we began with the simulated dataset of alcohol consumption trajectories. Each trajectory was truncated to include only the initial span (Day 1 only) of the developmental period, and data from all three subgroups (50 mice per subgroup) were then split into 70% training and 30% evaluation sets. Following this, one additional day of data was added to the truncated dataset at each step, a new split was independently generated, and the training and evaluation process was repeated. This iterative truncation continued until the full duration of the longitudinal dataset (Day 1–52) was completed.

We applied this approach to ML modeling by selecting twelve widely used ML algorithms based on a literature survey of reputable and effective supervised ML algorithms [54–56]. Further, a number of considerations were taken into account regarding algorithm characteristics. Firstly, when considering model structure and capacity, both linear models (Logistic Regression, Linear Discriminant Analysis) and nonlinear models (Decision Tree, Support Vector Machine) are represented. Also represented are parametric (Multinomial Naive Bayes, Logistic Regression) and non-parametric models (K-Nearest Neighbors, Random Forest, Gaussian Process Classifier). Secondly, when considering loss function and optimization approach, ML models include likelihood-based (Logistic Regression), margin-based losses (Support Vector Machine), and additive/ensemble optimization (Gradient Boosting Classifier). Lastly, when considering model interpretability, some models are highly interpretable (Logistic Regression, Linear Discriminant Analysis), while others are considered to have moderate or low interpretability (Random Forest, Multilayer Perceptron).

**Fig 3:**
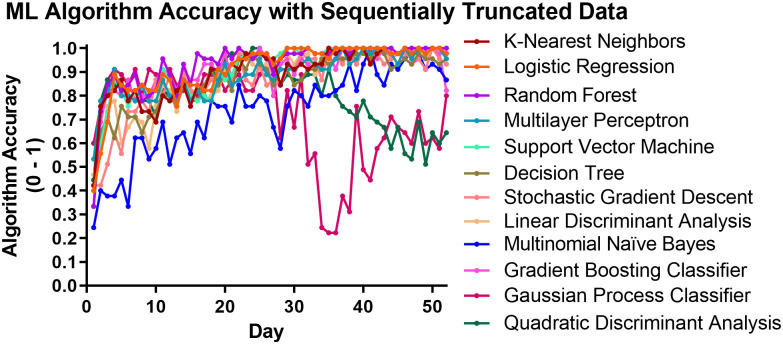
Distributions of prediction accuracy for trajectory subgroup classification for the twelve ML algorithms across sequentially truncated datasets. Each curve represents a different ML algorithm, showing how prediction accuracy for trajectory subgroup classification changes as additional days of data are included. Many, but not all, of the algorithms showed improved predictive performance as longer spans of behavioral data became available.

For each round of sequential dataset truncation, an ML model was trained on the training set, and then model performance was evaluated in terms of accuracy for trajectory subgroup classification on the held-out test set. The results of all twelve ML algorithms are displayed in Fig 3, with many of the algorithms showing improved predictive performance as longer spans of behavioral data became available.

### 3.3. Quantitative evaluation and comparison of ML algorithms for trajectory prediction accuracy

As prediction accuracy values showed day-to-day variability, LOESS [57] was applied to each model’s accuracy curve to smooth the results. This approach provides a smoothed trajectory of prediction accuracy over time, making it easier to extract meaningful trends and to perform direct comparisons among various ML models. MAE from the LOESS fit was used as a summary statistic for the various ML algorithms, and algorithm accuracy with LOESS fit data is provided in Supporting Information (S2 Data).

**Fig 4:**
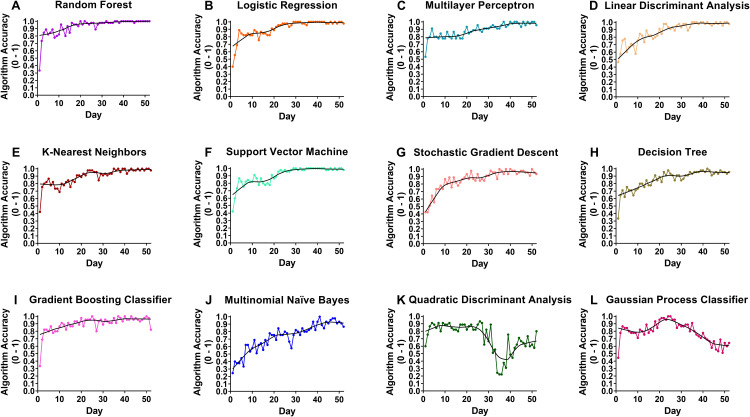
Prediction accuracy progression and LOESS-fitted trends for the twelve ML algorithms. Each panel (A–L) presents prediction accuracy values for trajectory subgroup classification (dots) for one ML algorithm over the experimental period, overlaid with a LOESS-fitted curve (solid black line).

Fig 4 presents the prediction accuracy distributions for each of the twelve ML models across experimental days, overlaid with LOESS-fitted curves. These smoothed fits illustrate each algorithm’s overall prediction behavior, capturing both early and late phase accuracy trends.

To establish a quantitative rubric for model evaluation, we focused on two metrics: prediction accuracy at the midpoint of the experiment (i.e., the first half of the 52 time points were available for training), and accuracy at the final timepoint. These values were extracted from the LOESS curves and tabulated in Table 1. This approach enabled numerical comparison of algorithms at key experimental points. Models are assigned to three categories based on their performance: “*outstanding*,” “*acceptable*,” and “*dreadful*.” The nomenclature of the categories was adapted from a widely known fictional grading system [66], and the numerical criteria for category assignments are detailed below.

**Table 1:** Goodness-of-fit for the prediction accuracy curves of ML algorithms.

Algorithm			Algorithm Accuracy (LOESS)	
Category	Full Name	Abbreviation	Midpoint	Experiment End
*Outstanding*	Random Forest	RF	0.97	1.00
	Logistic Regression	LR	0.97	0.99
	Multilayer Perceptron	MLP	0.90	0.98
	Linear Discriminant Analysis	LDA	0.93	0.99
	K-Nearest Neighbors	KNN	0.95	0.99
	Support Vector Machine	SVM	0.97	0.99
*Acceptable*	Stochastic Gradient Descent	SGD	0.88	0.95
	Decision Tree	DT	0.91	0.95
	Gradient Boosting Classifier	GBC	0.95	0.96
	Multinomial Naive Bayes	MNB	0.77	0.91
*Dreadful*	Quadratic Discriminant Analysis	QDA	0.82	0.67
	Gaussian Process Classifier	GPC	0.94	0.61

**Fig 5:**
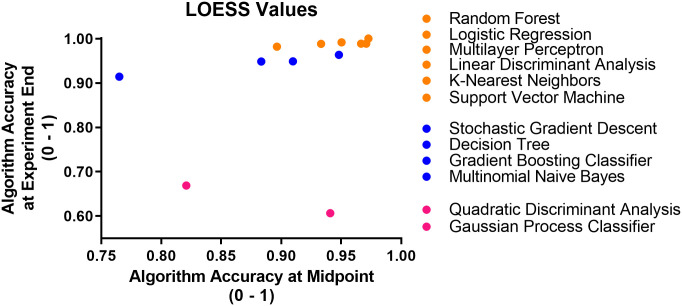
Comparison of midpoint and end-point prediction accuracy for twelve ML algorithms. Each dot represents one algorithm, plotted by its accuracy at the experiment midpoint (X-axis) and the final timepoint (Y-axis), as extracted from the LOESS-fitted curves. Algorithms are color-coded into three performance outcome-based categories: “*outstanding*” (gold) with high accuracy for trajectory subgroup classification, “*acceptable*” (blue) with moderate performance, and “*dreadful*” (red) with poor accuracy and a deteriorating tendency. This bivariate analysis offers a visual rubric for comparison across various ML algorithms.

Fig 5 shows a scatterplot of the midpoint versus the end-point accuracy for all twelve ML algorithms. We classified the algorithms into three performance outcome-based categories. Six algorithms—Random Forest, Logistic Regression, Multilayer Perceptron, Linear Discriminant Analysis, K-Nearest Neighbors, and Support Vector Machine—all achieved midpoint accuracies for trajectory subgroup classification of 90% or better, and end accuracies of 98% or better. These algorithms were deemed “*outstanding*.”

Four additional algorithms—Stochastic Gradient Descent, Decision Tree, Gradient Boosting Classifier, and Multinomial Naive Bayes—achieved acceptable accuracy values for trajectory subgroup classification (77–95% at midpoint, and 91–96% at experiment end), even though they did not meet the more stringent criteria of high end accuracy cutoff (98%) for the *outstanding* classification. These were therefore categorized as “*acceptable*.”

In contrast, two algorithms—Quadratic Discriminant Analysis and Gaussian Process Classifier—were assigned to the “*dreadful*” category. These algorithms exhibited both poor end-point accuracy values for trajectory subgroup classification (below 70%) and a troubling tendency of deteriorating performance with longer spans of training data, as visualized in Fig 4K and 4L, and quantified in Table 1.

## 4. Discussion

Adolescence is a developmental period marked by profound and stressful physical, behavioral, and cognitive changes [1–7]. Alcohol consumption during adolescence can lead to AUD later in life [8–18]. Studies have shown that innate processes such as genetics play a key role in AUD risk [26,29], as do the combined contributions of genetic, epigenetic, and environmental factors to addiction vulnerability [27,28]. Rodent models allow experimental studies of alcohol consumption behavior, including profiling cellular activities and markers in brain and other tissues that accompany a behavioral phenotype. However, there is the challenge of how to profile such activities and markers while the animals are still alive, so that key biological factors driving the subsequent behavior can be identified. That is, how to “predict the future” in animal research, while the behavioral phenotype is still being manifested.

One potential approach is to harness the power of computational algorithms for providing predictions of the eventual outcome of patterned behaviors, as ML offers a robust methodology for pattern recognition [38–40]. In the present study, we hypothesized that ML can be utilized with relatively limited biological data, for pattern recognition of voluntary alcohol consumption in adolescent mice. We evaluated ML algorithms for their ability at predicting the eventual trajectory of alcohol consumption behavior. Prediction accuracy was assessed with daily alcohol measurements from simulated mouse data with balanced classes, so that prediction accuracy values for each ML algorithm were obtained with increasing longer spans of truncated data (from the minimum on Day 1, to the end the experiment on Day 52).

Overall, the results supported our hypothesis. Notably, some of the *outstanding* ML algorithms were able to achieve 90% + prediction accuracy for trajectory subgroup classification even at the midpoint of the experiment, which is only halfway into the adolescent development period in mice. With longer spans of training data towards the end of the adolescence-to-adulthood development period, these *outstanding* ML algorithms were capable of achieving 98% or better prediction accuracy (Table 1, Fig 5). Such high levels of prediction accuracy offer a hitherto unavailable avenue in biological research: the ability to sample and analyze cellular activities and markers from mouse brains and other tissues—before the behavioral phenotype in an adolescent mouse is fully manifested. Such investigations may provide valuable insight into early biological processes, by sampling cellular and molecular biomarkers from the brain and somatic tissues, that may drive the subsequent alcohol consumption behavior during adolescence development, and contribute to better understanding of the fundamental molecular and neurobiological mechanisms that guide the onset of harmful alcohol consumption behavior in mice.

Not all tested ML algorithms achieve such high prediction accuracy for trajectory subgroup classification. Some ML algorithms produced acceptable accuracy for trajectory subgroup classification (between 91% and 96%) by experiment end, while two ML algorithms were unsuccessful at attaining high levels of accuracy (Table 1, Fig 5). As a side note, the combined visual and quantitative assessment framework (Fig 5) appears to offer a handy approach to evaluate ML algorithms for their ability to predict developmental trajectory-based behavioral patterns, using the example of voluntary alcohol consumption behavior in adolescent mice.

It should be pointed out that our study was based on one particular dataset of adolescent mouse behavior [33]. In particular, because two of the alcohol-consuming subgroups had very small numbers of biological samples from which the simulated datasets were generated, caution should be exercised when attempting to apply this strategy to other behavioral datasets. It is suggested that various ML algorithms be first tested with the intended behavioral dataset, so high accuracy ML algorithms can be selected that better suit the dataset at hand.

Particular caution is drawn to the fact that this is an exploratory study, based on relatively modest sample sizes (especially for the two mouse subgroups with high alcohol-consumption behavior). Using real-world longitudinal datasets with modest subgroup sizes is a key design feature for our study, for the following reasons. Firstly, the primary objective of this study is not to infer population-level parameters from small-sized sample sets, but rather to develop and evaluate an ML framework under realistic experimental conditions, where behavioral datasets are often imbalanced, and sample sizes within minority subtypes can be small. Such conditions are common in longitudinal behavioral studies, and more broadly in biomedical research. Therefore, the inclusion of small subgroups (n = 4–5 in the high alcohol-consumption behavior subgroups) is intentional, as it reflects the real-world situation of many discovery-oriented exploratory studies rather than a statistically fully powered and balanced dataset. Secondly, small sample sizes can be a feature of discovery-driven biomedical research, and important biological insights have often emerged from initial observations in small cohorts, which are subsequently validated in larger populations. Indeed, small-sample observations have historically played an important role in identifying novel phenotypes and disease entities, particularly in early-stage or hypothesis-generating research. For example, witness Alzheimer’s description of a new disease in a single patient [34,35] and Parkinson’s clinical description of a new disease based on six patients [36,37]. In our study, the small subgroup sizes are not used to make definitive population-level claims, but rather to evaluate whether ML approaches can remain informative under such constrained conditions. Thirdly, the purpose of simulated data in our study is not for biologically valid extrapolation of biological mechanism conclusions, but for stress-testing ML model behavior. Thus, it is important to note that the intent is not to reconstruct the “true” distribution of each subgroup, nor to imply that the simulated data fully represents biological variability. Rather, the intent is to provide controlled, reproducible inputs for evaluating ML algorithm performance, and to systematically assess robustness based on limited data. In this context, potential overfitting or sensitivity to small samples is precisely the phenomenon being evaluated, rather than an unintended artifact. Lastly, attention is drawn to the biological and translational relevance of the dataset. The dataset used in this study was selected because it captures behavioral trajectories that are consistent with known patterns in human alcohol use: one subgroup of mice exhibits early onset and sustained high consumption of alcohol, another subgroup exhibits gradual escalation over time, and a third majority group maintains a low consumption level over the entire duration. These voluntary alcohol consumption behaviors over the developmental course parallel human alcohol use trajectories observed in clinical and epidemiological studies: extensive studies indicate the complex developmental trajectories of human alcohol consumption behavior patterns, with some individuals exhibiting heavy alcohol involvement early in adolescence, others experiencing a more gradual escalation of alcohol consumption across adolescence and into adulthood [67–74], and others experiencing little to no adverse effects from their exposure to alcohol. In addition, the relative distribution of mouse subgroups is consistent with population-level observations in human: In the United States, approximately 11–14% of individuals meet criteria for Alcohol Use Disorder (AUD); global estimates similarly suggest that only a minority of the population exhibit alcohol use disorders. Thus, the predominance of non-drinker mice and the existence of smaller high-risk subgroups in our dataset are biologically meaningful, which may be of interest to readers in the alcohol study field.

In summary, the present study provides an example and exploratory approach for taking advantage of the pattern recognition abilities of ML algorithms to help predict the developmental trajectory of voluntary alcohol consumption behavior in adolescent mice, thus making it possible to analyze cellular activities and markers at earlier times that may drive the subsequent behavior, while the experimental animal is still developing its behavioral patterns.

## Supporting information

S1 File Supporting Information. (DOCX)

S1 Data Alcohol Consumption Data. (XLSX)

S2 Data ML Algos LOESS Fit. (XLSX)

S3 Data ML Models and Parameters. (TXT)
