# Supplementary material for: Evaluating machine learning algorithms at predicting developmental trajectories using sequential dataset truncation of voluntary alcohol consumption in adolescent mice
Source: PLoS One. 2026 Jun 22;21(6):e0352197. doi: 10.1371/journal.pone.0352197 (PMC13286189; doi:10.1371/journal.pone.0352197)
Supplement: S1 File — (DOCX) [file pone.0352197.s001.docx]

Supporting Information

Python code used in the study is available at GitHub:

<https://github.com/NathanYu129/DAMSEL-2/tree/main>

Daily alcohol consumption data:

Algorithm accuracy and LOESS fit:

ML models and Parameters:
